# Impacts of highway traffic exhaust in alpine valleys on the respiratory health in adults: a cross-sectional study

**DOI:** 10.1186/1476-069X-10-13

**Published:** 2011-03-04

**Authors:** Marianne E Hazenkamp-von Arx, Christian Schindler, Martina S Ragettli, Nino Künzli, Charlotte Braun-Fahrländer, Lee-Jane S Liu

**Affiliations:** 1 Swiss Tropical and Public Health Institute, Department of Epidemiology and Public Health, Socinstrasse 57, P.O. Box, CH-4002 Basel, Switzerland; 2 University of Basel, Switzerland; 3 Department of Environmental and Occupational Health Sciences, University of Washington, Seattle, WA, USA

## Abstract

**Background:**

Most studies having shown respiratory health effects from traffic exhaust were conducted in urban areas with a complex mixture of air pollution sources. This study has investigated the potential impact of traffic exhaust on respiratory symptoms among adults living along a Swiss alpine highway corridor, where traffic exhaust from the respective trans-Alpine highway is the predominate source of air pollution.

**Methods:**

In summer 2005, we recruited 1839 adults aged 15 to 70 from a random sample of 10 communities along the Swiss alpine highway corridors. Subjects answered a questionnaire on respiratory health (asthmatic and bronchitic symptoms), risk factors, and potential confounding variables. We used logistic regression models to assess associations between respiratory symptoms and traffic exposure being defined a) as living within 200 m of the highway, and b) as a bell-shaped function simulating the decrease of pollution levels with increasing distance to the highway.

**Results:**

Positive associations were found between living close to a highway and wheezing without cold (OR = 3.10, 95%-CI: 1.27-7.55) and chronic cough (OR = 2.88, 95%-CI: 1.17-7.05). The models using a bell-shaped function suggested that symptoms reached background levels after 400-500 m from the highway. The association with chronic cough was driven by a subgroup reporting hay fever or allergic rhinitis.

**Conclusions:**

Highway traffic exhaust in alpine highway corridors, in the absence of other industrial sources, showed negative associations with the respiratory health of adults, higher than those previously found in urban areas.

## Background

Previous epidemiological studies on air pollution in urban regions showed negative associations between traffic-related pollution and respiratory health [1-10]. Most of these studies focused on the health of children [1-8], while few studied adults [9,10]. A major challenge of these studies is the assessment of long-term exposure to traffic-related pollutants [11]. Concentrations of these pollutants are usually several fold higher along busy traffic corridors than some 50-200 m away [12,13] and some components, such as ultrafine particles, have been identified as highly oxidative toxicants [14].

These spatial characteristics would call for dense measurements of primary particles. Instead for reasons of feasibility, studies often categorized distance between home and closest major road as a marker of exposure [2-7,15,16].

Often, only a dichotomous variable with a cutpoint between 50 and 300 m was considered [2,3,6,7,16-21] while other studies quantified traffic-related pollutants using a spline function of distance to the nearest major road [7] or estimated pollution levels using distance as one of the main linear predictor variables ([22,23], LJS Liu, submitted manuscript, 2010). As most urban study areas are characterized by both heavy traffic and high or complex background air pollution [2,3,16], the distance to the closest major road has been identified as an imprecise measure of exposure in complex urban settings [11].

However, in the simple case of a single dominant traffic artery, distance from this artery and wind direction become the primary determinants of local spatial contrasts in traffic-related pollutants. Gaussian dispersion theory suggests that, in such cases, exposure to traffic pollutants can be approximately described by a bell-shaped function of distance to the predominant traffic line source [24,25].

Switzerland provides one of the major trans-alpine routes for freight traffic, with one million trucks per year or 20,000-45,000 vehicles/day with 10% diesel truck traffic [26]. Many small towns are lined up along this artery, with distance to the respective highway expected to be clearly the dominant determinant of local contrasts in ambient concentrations of traffic-related emissions and resuspensions [26,27].

This study was nested in a socio-demographic study [28] involving a random sample of adults residing in 10 rural and alpine communities along the afore mentioned major highway as well as a secondary trans-alpine highway. We hypothesized that adults living closer to a highway are more likely to suffer from respiratory symptoms and that effects of traffic exhaust might be even more clearly visible in this sample than in a previous Swiss study involving both rural and urban areas with more complex structures and sources of pollution [9].

## Methods

### Study population

**Figure 1:**
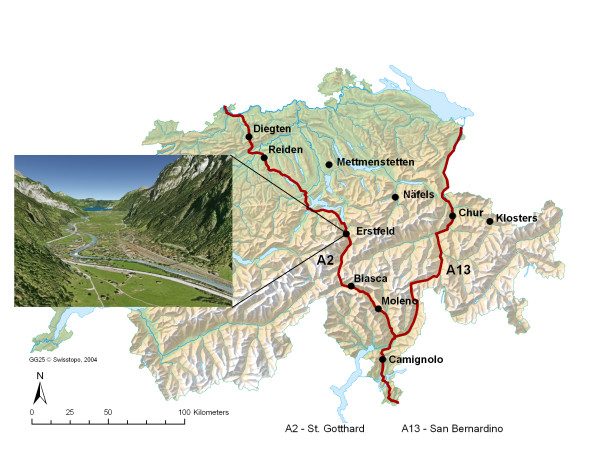
**Study area**. Map of Switzerland with the 10 study communities. The inset shows the topography in Erstfeld having a width of 800 m at the bottom of the valley.

**Figure 2:**
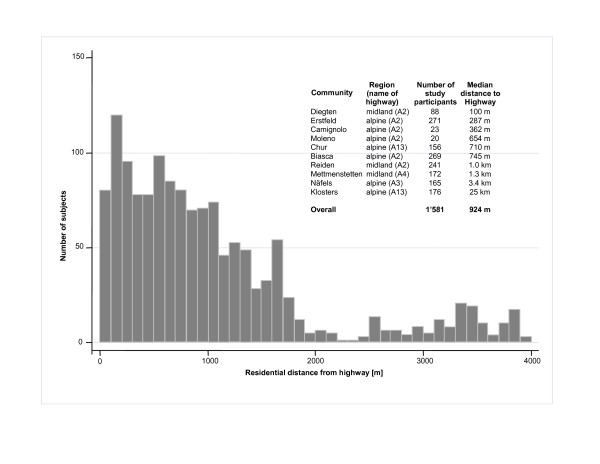
**Distribution of residential distances from highway for the MfM-U study population in 2005**. The histogram shows data from subjects living within 4 km of the highway.

Ten trans-alpine rural communities were selected to represent different climatic and topographic conditions (Figure 1) and to cover a broad range of distances to highways (Figure 2). Seven communities were situated in the alpine and three in the flatter midland region (Diegten, Reiden, Mettmenstetten). All communities with the exception of Klosters (a control) were situated within 4 km of the highway, with 50% of the participants living within 1 km of it. More than 99% of the study population lived at the bottom of the valleys at altitudes no more than 100 m above/below the highway. The width of the bottom of the valleys ranged between 0.6 and 2 km (see Figure 1 for example). One midland community was not located in a valley (Mettmenstetten). The population of most communities ranged between 1,200 and 5,800, except for Chur (pop = 33,000), Camignolo, and Moleno (pop <500 each).

Authorities of the communities provided the addresses and telephone numbers of residents aged between 15 and 70 years and a list of streets mostly affected by traffic. 3,287 persons were randomly selected from the address list in anticipation of a 60% response rate. 1,839 persons (or 56% response rate) answered the questionnaire during telephone interviews conducted by a professional poll company (DemoScope, Research and Marketing, Adligenswil, Switzerland) which engaged 78 interviewers between 6/30/2005 and 8/15/2005. Residents living close to a highway were over-sampled to increase the variation of exposure and statistical power. 49 participants were excluded due to insufficient address information and an additional 209 were excluded due to incomplete data.

### Health assessment/questionnaire data

The telephone interview was conducted for a socio-demographic survey in the first place but also included a condensed version of a questionnaire previously established in a Swiss air pollution cohort study [29] (See additional file 1: Health part of the questionnaire). This additional questionnaire focused on the history of respiratory symptoms, family history of disease, socio-economic status, smoking habits, environmental tobacco smoke (ETS), living and working environments, and residential situations. The following respiratory health outcomes were considered: wheezing with breathing problems in the last 12 months, wheezing without cold in the last 12 months, regular cough, regular phlegm, chronic cough and chronic cough or phlegm. Precise definitions of respiratory health outcomes are given in the additional file 2.

### Individual exposure assessment

Participants' addresses were geo-coded by matching to the building registry of the Swiss Federal Statistical Office or manually using Twixroute (version 11.2005, Twix AG, Egg, Switzerland). Only geo-coded addresses which could be matched at the house number level were used. The distances from the residential coordinates to the closest highway were computed based on the Swiss map VECTOR25 (Swiss Federal Office of Topography, Wabern, Switzerland). Based on the literature [2,3,6,7,16-21], we first used a dichotomous exposure variable for living within 200 m of a highway (typically >20,000 cars/day). We also defined an indicator variable for living within 50 m of a major local street (i.e., Class 1 major road at least 6 m wide, and typically <10,000 cars/day) to control for local traffic exhaust. To control for general background pollution, we used estimates of average PM_10 _levels outside subjects' residences from the PolluMap dispersion model for the year 2000 which is described in detail in Liu et al. [30]. Note that even the corresponding dispersion model for NO_2 _lacked sufficient spatial resolution for a valid estimation of traffic-related pollutants from local sources [LJS Liu, submitted manuscript, 2010]. Regulatory air measurements were not used as they were sparse and collected near highways.

We further replaced the binary variable for traffic exposure with a smooth bell-shaped function of the distance between the residence and the highway of the form i.e.,

$$C\left(d\right)=e^{-\frac{d^{2}}{2\sigma_{d}{}^{2}}}$$

The parameter σ_d _defines the distance of the two inflection points of the bell curve from the highway. Assuming the logit of the prevalence of a given respiratory symptom to be proportional to C(d), the parameter σ_d _can be estimated by iteratively fitting logistic regression models with stepwise improved values of σ_d _until a minimum of the likelihood function is reached. The parameter σ_d _may vary between symptoms if these are differently affected by traffic air pollution.

### Statistical Analysis

We used logistic regression to model the relation between residential exposure to air pollution from highway traffic and the prevalence of respiratory symptoms in our 1,581 study participants. Because of the low prevalence rates of certain symptoms, we used backward selection to limit the number of model covariates, which were identical to those in Bayer-Oglesby et al. [9] at the start. Covariates were eliminated if they did not improve predictions (AIC criterion) and if the odds ratio of the exposure variable "living within 200 m" did not change by more than 0.05 upon their removal.

Our final model included sex, age, smoking status (current, former, never), pack years of cigarettes smoked, body mass index, community of residence and the binary variables exposure to environmental tobacco smoke (ETS), ETS-exposure at work, current occupational exposure to vapors, gas, dust, fumes, or aerosols, primary school education only, doctor diagnosed asthma, maternal atopy, and severe respiratory infection in early childhood. "Doctor diagnosed asthma" was included in the model as there was some indication that people with asthma tended to live farther away from the highway. Based on published results indicating particular susceptibility to air pollution among persons with respiratory allergies [31-33], we also examined whether associations with traffic exposure differed between subjects with and without allergic rhinitis by introducing an interaction term between allergic rhinitis and traffic exposure. If there was evidence of an interaction, we used separate traffic exposure terms for the two subject groups to obtain specific estimates for each of the two sub-samples. The parameter σ_d _in equation (1) was determined by maximizing the likelihood of the respective logistic regression model. Sensitivity analyses were performed to test the stability of results upon exclusion of single communities, subjects with primary school education only (n = 90), older age (age > 60, n = 266), or manually geo-coded addresses (n = 61). All analyses were performed with the statistical software STATA/SE 9.1 (StataCorp LP, College Station, TX USA, StataCorp LP, 2006).

## Results

Table 1 shows characteristics of all participants and a subgroup with allergy responses, grouped by exposure levels. The prevalence of respiratory symptoms ranged from 5% for wheezing with breathing problems, 15% for regular cough to 21% for allergic rhinitis or hay fever. The high exposure group reported more wheezing, chronic cough, and chronic cough or phlegm and slightly more regular phlegm, regular cough, and allergic rhinitis or hay fever. However, the prevalence of doctor diagnosed asthma was lower in this group.

**Table 1:** Characteristics of the study population and a sub-group with allergy by residential distance to highway.

	All participants (total group)	Low exposure	High exposure	Low exposure	High exposure
		
		(>200 m of a highway)	(≤200 m of a highway)	Participants with allergic rhinitis or hay fever	
	n = 1581	n = 1384	n = 197	n = 288	n = 42

Men (%)	46.5	45.6	52.8	46.9	57.1

Age, mean	41.7	41.9	40.2	37.7	36.5

Primary school education only (%)	5.7	5.7	5.6	3.8	2.4

Swiss nationality (%)	88.2	87.6	92.4	88.2	90.5

Body Mass Index, mean	24.4	24.4	24.5	23.7	24.2

Maternal atopy (%)	12.4	12.9	8.6	25.7	9.5

Early childhood respiratory infection (%)	7.7	7.4	9.6	11.5	11.9

ETS exposure (%)	27.3	27.0	28.9	30.2	31.0

ETS exposure at workplace (%)	12.5	12.6	11.7	13.5	11.9

Occupational exposure, current (%)	12.7	12.5	13.7	11.1	16.7

Never smoker (%)	57.2	57.6	54.8	63.9	71.4

Current smoker (%)	22.6	22.3	24.9	17.7	11.9

Former smoker (%)	20.2	20.2	20.3	18.4	16.7

Smoked cigarettes (packyears), mean	6.6	6.6	6.7	4.3	6.0

Wheezing with breathing problems (%)	4.7	4.5	6.1	9.7	11.9

Wheezing without colds (%)	4.9	4.6	7.1	7.3	11.9

Regular phlegm (%)	10.5	10.3	11.7	13.2	16.7

Regular cough (%)	15.0	14.9	15.2	18.9	21.4

Chronic cough (%)	5.8	5.4	8.1	6.6	16.7

Chronic cough or chronic phlegm (%)	6.7	6.5	8.1	9.4	16.7

Doctor diagnosed asthma (%)	8.9	9.1	7.1	20.8	7.1

Allergic rhinitis or hay fever (%)	20.9	20.9	21.3		

A more complete table with n's is given in the Additional File 3 (Table S1).

Participants lived at a median distance of 924 m to the respective highway, with 12.5% (n = 197) living within 200 m of it (Figure 2). High proportions of participants lived close to the highway in Diegten (92%) and Erstfeld (34%), whereas very few or no participants lived close to the highway in Biasca, Reiden, Mettmenstetten Näfels, and Klosters. About 28% (n = 445) of the participants lived within 50 m of a major street.

Estimated regional background PM_10 _concentrations from the dispersion model [30] ranged between 9 and 10 μg/m^3 ^for communities in the North, between 16 and 18 μg/m^3 ^in the South of Switzerland, and between 16 and 26 μg/m^3 ^outside subjects' residences. Available PM_10 _measurements at five highway locations in the study region ranged between 22 and 29 μg/m^3^[26].

Table 2 shows increased adjusted odds of reporting respiratory symptoms for those living close to a highway. Strongest associations were found for wheezing with breathing problems (OR = 2.64), wheezing without colds (OR = 3.10), and chronic bronchitis symptoms including chronic cough (OR = 2.88) or phlegm (OR = 2.4). But the odds of reporting regular cough and regular phlegm were also slightly elevated.

**Table 2:** Adjusted OR and 95%CI of reported respiratory symptoms for "living within 200 m of a highway*.

Symptoms	Total group n = 1566-1568	Participants with or without hay fever or allergic rhinitis n = 1561-1563		
		
		with hay fever or allergic rhinitis	without hay fever or allergic rhinitis	p-value for interaction
Wheezing with breathing problems	2.64 (1.07-6.48)	2.91 (0.76-11.2)	2.42 (0.86-6.81)	0.80

Wheezing without colds	3.10 (1.27-7.55)	4.64 (1.22-17.7)	2.63 (0.98-7.04)	0.43

Regular cough	1.36 (0.72-2.56)	1.89 (0.72-5.01)	1.27 (0.64-2.52)	0.43

Regular phlegm	1.19 (0.60-2.38)	1.41 (0.49-4.06)	1.09 (0.52-2.31)	0.65

Chronic cough	2.88 (1.17-7.05)	7.14 (2.06-24.7)	2.01 (0.7-5.46)	0.048

Chronic cough or phlegm	2.40 (1.01-5.70)	4.73 (1.43-15.7)	1.78 (0.6-4.69)	0.12

*as traffic exposure proxy.

The adjustments include sex, age, smoking status (current, former, never), pack years of cigarettes smoked, body mass index, community of residence and the binary variables exposure to environmental tobacco smoke (ETS), ETS-exposure at work, current occupational exposure to vapors, gas, dust, fumes, or aerosols, primary school education only, doctor diagnosed asthma, maternal atopy, and severe respiratory infection in early childhood.

Whereas inclusion of regional background PM_10 _estimates had almost no effects, exclusion of participants living within 50 m of a major street, resulted in stronger associations with highway exposure for cough and phlegm symptoms but weaker associations for the wheezing symptoms. We thus adjusted for "living within 50 m of a major street" in all our final models.

**Figure 3:**
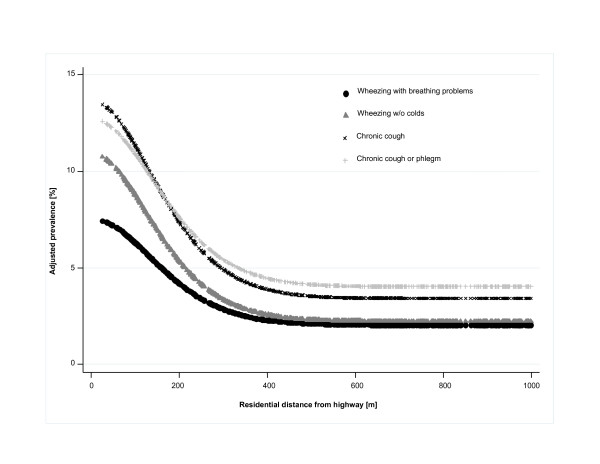
**Estimated adjusted prevalence rates of reported health outcomes**. The prevalence rates are displayed as bell-shaped functions of the residential distance from highway [m]. The underlying logistic regression models included sex, age, smoking status (current, former, never), pack years of cigarettes smoked, body mass index, community of residence and the binary variables exposure to environmental tobacco smoke (ETS), ETS-exposure at work, current occupational exposure to vapors, gas, dust, fumes, or aerosols, primary school education only, doctor diagnosed asthma, maternal atopy, and severe respiratory infection in early childhood.

The parameter σ_d _of the smooth exposure function in the logistic regression model for the most affected symptom, "wheezing without colds" was 180 m. Interestingly, σ_d _was similar for other reported symptoms. Figure 3 shows the adjusted prevalence rates of different respiratory symptoms as a function of the residential distance from the highway. Chronic cough and chronic cough/phlegm had a similar background prevalence at residential distances beyond 500 m from the highway; however, the prevalence of chronic cough had a steeper incline with decreasing distance to the highway. Similarly, wheezing without colds had almost the same background prevalence as wheezing with breathing problems but increased faster in proximity to the highway.

As shown in Table 1, the increase in the risk of chronic cough with living close to a highway was even stronger among participants reporting allergic rhinitis or hay fever. This is confirmed by the interaction analysis where the 95%-confidence interval of the odds ratio exceeds 2 in the respective subgroup (Table 2). Also for the other symptoms, the rise in prevalence with decreasing distance to the highway was slightly stronger in this subgroup. Estimated associations between symptoms and exposure to highway traffic were only slightly weakened when excluding asthmatic subjects (see additional file 3: Table S2 that reports adjusted odds ratios and 95% confidence intervals for reported respiratory symptoms in participants with and without asthma).

### Sensitivity and supplementary analyses

The odds ratios of wheezing without colds or chronic cough did not change when midland communities (Diegten, Mettmenstetten, and Reiden) were excluded. Alternating exclusion of one community at a time did not lead to relevant changes in the odds ratio of chronic cough. However, the exclusion of Erstfeld with its relatively large proportion of subjects living close to the highway weakened the result for wheezing without colds. Results of further sensitivity analyses are provided in the additional file 3. This supplement includes a table (Additional file 3: Table S3) with odds ratios only adjusted for community but not for individual factors, a table (Additional file 3: Table S4) with odds ratios unadjusted for doctor's diagnosed asthma and tables with results obtained after exclusion of non-Swiss citizens (S5) or participants living within 50 m of a major road (S6).

## Discussion

This study provides evidence that residential exposure to highway traffic increases respiratory symptoms in rural regions with no major other local sources. The strongest associations found were for asthma-related symptoms including wheezing without a cold and wheezing with breathing problems, as well as for bronchitic symptoms including chronic cough and chronic cough/phlegm.

Our findings are consistent with results from previous epidemiologic studies linking traffic exposures in urban areas with wheezing [9,18], bronchitic symptoms [9,18], or chronic bronchitis [10,19,34] in adults, and wheezing [2-7,15,16], cough [4,5,16,20], and bronchitis [20] in children. However, for most of the symptoms considered, our observed odds ratios were considerably higher than those reported for adults living close to major roads in studies from other countries [10,18,19,34]. Comparable odds ratios (i.e., between 2 and 3) were so far only reported for children [2-7,15,16], and in our own previous study in Switzerland [9]. In our study communities, highway traffic emissions were the predominant pollution source, with EC measurements next to these highways reaching 4-9 μg/m^3 ^[26] as compared with those in rural (0.5-1 μg/m^3^) and urban areas (~2 μg/m^3^) without a highway [27]. This contrasts the more complex pollutant characteristics in urban areas, where a multitude of local street systems and other emissions may result in substantial exposure misclassification if 'distance' is used as the only marker of exposure. In such settings, effects from traffic emissions or resuspensions thus tend to be obscured and biased toward null findings [e.g [35]].

Unlike for the highway, we found no effects from rural major roads (traffic volume mainly <10,000 cars/day) suggesting a primary role of heavy duty (diesel) traffic which mostly concentrates on the highways while local streets are mostly used by cars. The proportion of Diesel passenger cars is still very low in Switzerland.

We observed a stronger association between chronic cough and distance to highway among subjects with hay fever or allergic rhinitis. This is consistent with subgroup results from other observational studies in children [33] and adults [31,32] and from experimental studies [36] that exposed subjects to diesel particles and it adds to the evidence that persons with respiratory allergies are more susceptible to the effects of air pollution. However, the prevalence of asthma was not affected by proximity to highway which contrasts with other studies and in particular the recent findings of the SAPALDIA study with adult onset asthma being associated with modeled traffic-related particulate matter [37]. However, this SAPALDIA investigation was longitudinal and targeted incidence while our project assessed cross-sectional associations. Our design may be affected by self-selection if asthmatics were to change their residence in relation to environmental exposures. We have no data to elaborate on this. However, SAPALDIA did indeed confirm that asthmatics were prone to choose the place of living due to their disease [38].

Consistent results were obtained with two different indices for traffic exposure. The bell-shaped functions provided quantitative and visual descriptions of the relationship between the prevalence of respiratory symptoms and residential distance to the highway. Such dose-response curves (Figure 3) provide specific risk estimates at different residential locations. The distance from the highway where the prevalence of symptoms started to level off was between 400-500 m which agrees with previous findings from Gauderman et al. [8], Gilbert et al.[39] and Jerrett et al. [40], reporting a leveling off of e.g. NO_2 _concentrations not until several kilometers from the highway. Moreover, the standard deviation (180 m) of the bell-shaped dose-response curves appeared to be approximately the same for all symptoms and communities. Our findings suggest that regardless of differences in etiology and relevant pollutant components (be that soot, ultrafine particles, organic carbon, metals, NO_2_, etc.), the risk gradients might be remarkably similar for different respiratory symptoms.

This stable standard deviation of ~180 m also justified the threshold of 200 m used to define the binary exposure variable used in our models. As the binary index of exposure does not capture the actual dispersion profiles, the resulted odds ratios provided only a simple risk stratification. In contrast, the bell-shaped function can provide detailed information on the decrease in risk with growing residential distance to the highway. Most studies having looked at health outcomes in relation to distance to major roads did not take into account prevailing wind directions and used the same distance thresholds for both sides of a major road. We did the same, but given that most of our study areas were located in alpine valleys, prevailing wind directions were essentially parallel to the highway.

In our study, the participation rate was 56% which is comparable to participation rates of previous studies involving telephone interviews, i.e., 58% in the U.S. Veteran study [18], and 61% in the National German Health Survey [34]. To examine potential participation bias, we compared the distances of participants' and non-participants' homes to the highway but did not find any systematic differences. In particular, the percentage of persons living within 200 m of a highway was almost identical in participants and non-participants. In a subsample of 797 participants we had additional information on the satisfaction with air quality expressed as a score. In this subsample, effect estimates for traffic exposure were very similar with and without adjustment for this score (see additional file 3: Sensitivity and supplementary analyses, Table S7). This argues against strong reporting bias. Also, reporting bias might be less of concern among subjects with nasal allergies as these people are likely to be more aware of respiratory symptoms.

Moreover, symptom prevalence and smoking rates in our sample were comparable to those observed in the SAPALDIA-study [9,29], i.e., in a representative population sample of adults in Switzerland.

A major limitation of our study is its limited sample size due to the sparse population in the Alpine highway corridors. Another limitation is the lack of data on ambient or home outdoor pollution measurements, and the limited information on the subjects' workplace.

## Conclusions

Our findings add to the growing evidence that exposure to traffic-related pollutants concentrated along busy roads is associated with increased risks of respiratory symptoms in adults. Most previous studies addressed such issues in urban settings. This study presents the first epidemiological results on traffic pollution effects in rural Alpine communities with one prevailing emission source (highway). We used a bell-shaped function of distance to highway to overcome the inherent arbitrariness in defining a binary distance index and to explore the profiles of risk gradients. Our study further indicates that subjects with nasal allergies are more vulnerable to traffic air pollution from a highway. Further research is needed to identify particularly harmful components of traffic exhaust. Ideally, such studies should be performed in a similar setting, but they ought to include larger populations, objective health measurements and spatially resolved exposure measurements.

## Abbreviations

AIC: Akaike information criterion; ETS: environmental tobacco smoke; FOEN: Federal Office of the Environment, Switzerland; NO_2_: nitrogen dioxide; OR: odds ratio; PM: particulate matter; PM_10_: particulate matter with aerodynamic diameter less than 10 μm; Pop: Population; 95%CI: 95% confidence interval.

## Competing interests

The authors declare that they have no competing interests.

## Authors' contributions

MEH, CBF, CS, LJSL and NK have made substantial contributions to conception and design of the study. MEH and CBF have been concerned with the acquisition of data. All authors have made substantial contributions to the analysis and/or interpretation of the data. All authors have been involved in drafting the manuscript and/or revising it critically for important intellectual content. All authors read and approved the final manuscript.

## Supplementary Material

Additional file 1 **Health part of the questionnaire**. PDF file that shows the questionnaire (health part) of the telephone interview. Click here for file

Additional file 2 **Definitions of reported respiratory symptoms**. Text document that provides precise definitions of respiratory health outcomes. Click here for file

Additional file 3 **Sensitivity and supplementary analyses**. This document provides a more complete version of Table 1, additionally containing the n's of the different categories (Table S1). In addition, it contains six tables with results from supplementary analyses on the association between reported respiratory symptoms and "living within 200 m of a highway": Table S2 reports adjusted odds ratios for reported respiratory symptoms in participants with and without asthma. Table S3 reports odds ratios adjusted only for community but not for individual factors. Table S4 shows odds ratios unadjusted for doctor's diagnosed asthma. Table S5 shows the results obtained after exclusion of non-Swiss citizens. Table S6 reports odds ratios for participants living more than 50 m from a major road. Finally, Table S7 reports odds ratios adjusted for satisfaction with air quality. Click here for file
