# Supplementary material for: Impacts of highway traffic exhaust in alpine valleys on the respiratory health in adults: a cross-sectional study
Source: Environ Health. 2011 Mar 4;10:13. doi: 10.1186/1476-069X-10-13 (PMC3059289; doi:10.1186/1476-069X-10-13)
Supplement: Additional file 1 — Health part of the questionnaire. PDF file that shows the questionnaire (health part) of the telephone interview. [file 1476-069X-10-13-S1.PDF]

Additional file 1: Health part of the questionnaire

MfM-U Codierung	SAPALDIA -Codierung	Frage
32	T_H02260	Q20. Wie stark stört Sie die **Luftverschmutzung** zu Hause, d.h. der Gestank von Verkehr und Industrie, wenn Sie das Fenster geöffnet haben?<br>Skala (0 - 10) Grad der Störung<br><br>_0: stört kein bisschen<br>_1<br>_2<br>_3<br>_4<br>_5<br>_6<br>_7<br>_8<br>_9<br>_10: stört unerträglich<br>_98 WEISS NICHT<br>_99 KEINE ANTWORT
33	T_H02250	Q21. Wie stark stört Sie der **Verkehrslärm** zu Hause, wenn Sie das Fenster geöffnet haben?<br>Skala (0 - 10) Grad der Störung<br><br>_0: stört kein bisschen<br>_1<br>_2<br>_3<br>_4<br>_5<br>_6<br>_7<br>_8<br>_9<br>_10: stört unerträglich<br>_98 WEISS NICHT<br>_99 KEINE ANTWORT

34	G_1	Q22. Welche Lärmquelle ist für Sie die Hauptquelle der Lärmstörung?<br><br>_1 Autobahn<br>_2 Hauptstrasse<br>_3 Quartierstrasse<br>_4 Eisenbahn<br>_98 WEISS NICHT<br>_99 KEINE ANTWORT
35	G_2	Q23. Wie gross sind Sie? (z.B. Info aus Pass)<br><br>_________ cm (Zahl einfüllen)<br>_98 WEISS NICHT<br>_99 KEINE ANTWORT

36	G_3	Q24. Wie schwer sind Sie?<br><br>_________ kg (Zahl einfüllen)<br>_98 WEISS NICHT<br>_99 KEINE ANTWORT
37.	T_H00010	Q25. Haben Sie in den letzten 12 Monaten irgendwann ein pfeifendes Atemgeräusch in der Brust gehabt?<br><br>_1 nein → *gehen Sie bitte zu Frage T_H00050*<br>_2 ja<br>_98 WEISS NICHT<br>_99 KEINE ANTWORT
38.	T_H00020	Q26. Haben Sie in den letzten 12 Monaten Mühe gehabt mit Atmen, wenn Sie dieses pfeifende Atemgeräusch in der Brust gehabt haben?<br><br>_1 nein<br>_2 ja<br>_98 WEISS NICHT<br>_99 KEINE ANTWORT
39.	T_H00030	Q27. Haben Sie in den letzten 12 Monaten dieses pfeifende Atemgeräusch gehabt,<br>ohne dass Sie gleichzeitig erkältet waren?<br><br>_1 nein<br>_2 ja<br>_98 WEISS NICHT<br>_99 KEINE ANTWORT
40.	T_H00050	Q28. Haben Sie in den letzten 12 Monaten tagsüber einen Anfall von Atemnot gehabt, wenn Sie ruhig waren? (gemeint ist "in Ruhe")<br><br>_1 nein<br>_2 ja<br>_98 WEISS NICHT<br>_99 KEINE ANTWORT
41.	T_H00070	Q29. Sind Sie in den letzten 12 Monaten jemals aufgewacht, weil Sie plötzlich Atemnot gehabt haben?<br><br>_1 nein<br>_2 ja<br>_98 WEISS NICHT<br>_99 KEINE ANTWORT

42.	T_H00110	Q30. Husten Sie **normalerweise** morgens nach dem Aufstehen?<br><br>_1 nein<br>_2 ja<br>_98 WEISS NICHT<br>_99 KEINE ANTWORT
43.	T_H00130	Q31. Husten Sie **normalerweise** tagsüber oder nachts?<br><br>_1 nein → *wenn T_H00110 „nein“* ***und*** *T_H00130 „nein“ gehen Sie bitte zu Frage T_H00170*<br>_2 ja<br>_98 WEISS NICHT

		_99 KEINE ANTWORT
44.	T_H00140	Q32. Husten Sie so an den meisten Tagen während mindestens 3 Monaten im Jahr?<br><br>_1 nein<br>_2 ja<br>_98 WEISS NICHT<br>_99 KEINE ANTWORT
45.	T_H00160	Q33. Seit wie vielen Jahren?<br><br>*Falls keine Antwort, neu fragen:*<br>*2. Versuch:* *Seit wie vielen Jahren husten Sie **mindestens** so?*<br>*3. Versuch:* *Husten Sie seit **mindestens 2 Jahren** so?*<br><br>______(Zahl einfüllen)<br>_98 WEISS NICHT<br>_99 KEINE ANTWORT
46.	T_H00170	Q34. Haben Sie normalerweise Auswurf morgens nach dem Aufstehen?<br><br>_1 nein<br>_2 ja<br>_98 WEISS NICHT<br>_99 KEINE ANTWORT

47.	T_H00190	Q35. Haben Sie normalerweise tagsüber oder nachts Auswurf?<br><br>_1 nein *→ wenn T_H00170 „nein“ **und** T_H00190 „nein“ gehen Sie bitte zu Frage T_H00310*<br>_2 ja<br>_98 WEISS NICHT<br>_99 KEINE ANTWORT
48.	T_H00200	Q36. Haben Sie normalerweise an den meisten Tagen während mindestens 3 Monaten pro Jahr solchen Auswurf?<br><br>_1 nein<br>_2 ja<br>_98 WEISS NICHT<br>_99 KEINE ANTWORT
		Q37. Seit wie vielen Jahren?<br><br>*Falls keine Antwort, neu fragen:*<br>*2. Versuch:* *Seit wie vielen Jahren haben Sie diesen Auswurf?*<br>*3. Versuch:* *Haben Sie diesen Auswurf seit **mindestens 2 Jahren** so?*<br><br>______(Zahl einfüllen)<br>_98 WEISS NICHT<br>_99 KEINE ANTWORT

49.	T_H00310	Q38. Haben Sie jemals Asthma gehabt?<br><br>_1 nein → *gehen Sie bitte zu Frage S266*<br>_2 ja<br>_98 WEISS NICHT<br>_99 KEINE ANTWORT
50.	T_H00320	Q39. Wurde dies von einem Arzt bestätigt?<br><br>_1 nein<br>_2 ja<br>_98 WEISS NICHT<br>_99 KEINE ANTWORT
51	T_H00370	Q40. Haben Sie in den letzten 12 Monaten einen Asthmaanfall gehabt?<br><br>_1 nein<br>_2 ja<br>_98 WEISS NICHT<br>_99 KEINE ANTWORT
52.	T_H00430	Q41. Nehmen Sie zur Zeit irgendwelche Medikamente gegen Asthma (auch Inhalationsmittel, Aerosole oder Tabletten)?<br><br>_1 nein<br>_2 ja<br>_98 WEISS NICHT<br>_99 KEINE ANTWORT

53.	S266	Q42. Nehmen Sie täglich Medikamente zur Verbesserung der Atmung, auch wenn Sie nicht Mühe haben mit der Atmung?<br><br>_1 nein<br>_2 ja<br>_98 WEISS NICHT<br>_99 KEINE ANTWORT
54.	T_H00500	Q43. Haben Sie allergischen Schnupfen oder Heuschnupfen?<br><br>_1 nein<br>_2 ja<br>_98 WEISS NICHT<br>_99 KEINE ANTWORT
55	T_H00741	Q44. Haben Sie etwas von dem Folgenden?<br><br>Hoher Blutdruck<br><br>_1 nein<br>_2 ja, aber nicht vom Arzt diagnostiziert<br>_3 ja, vom Arzt diagnostiziert<br>_98 WEISS NICHT<br>_99 KEINE ANTWORT

56.	T_H00745	Q45. Herzkrankheiten<br><br>_1 nein<br>_2 ja, aber nicht vom Arzt diagnostiziert<br>_3 ja, vom Arzt diagnostiziert<br>_98 WEISS NICHT<br>_99 KEINE ANTWORT
57.	T_H00748	Q46. Migräne/oft auftretende<br><br>_1 nein<br>_2 ja, aber nicht vom Arzt diagnostiziert<br>_3 ja, vom Arzt diagnostiziert<br>_98 WEISS NICHT<br>_99 KEINE ANTWORT
58.	T_H01000	Q47. Haben Sie jemals in einem Beruf gearbeitet, bei dem Sie Dampf, Gas, Staub, Rauch oder Aerosolen ausgesetzt waren?<br>*(nicht Zigarettenrauch)*<br><br>_1 nein<br>_2 ja<br>_98 WEISS NICHT<br>_99 KEINE ANTWORT
59	G_4	Q48. Arbeiten Sie heute immer noch in einem Beruf, bei dem Sie Dampf, Gas, Staub, Rauch oder Aerosolen ausgesetzt sind?<br>*(nicht Zigarettenrauch)*<br>_1 nein<br>_2 ja<br>_98 WEISS NICHT<br>_99 KEINE ANTWORT

60.	S064	Q49. Hat Ihre Mutter jemals Asthma gehabt?<br><br>_1 nein<br>_2 ja<br>_98 WEISS NICHT<br>_99 KEINE ANTWORT
61.	S065	Q50. Hat Ihre Mutter jemals Ekzeme, Hautallergien oder allergischen Schnupfen wie z.B. Heuschnupfen gehabt?<br><br>_1 nein<br>_2 ja<br>_98 WEISS NICHT<br>_99 KEINE ANTWORT
62	S066	Q51. Hat Ihr Vater jemals Asthma gehabt?<br><br>_1 nein<br>_2 ja<br>_98 WEISS NICHT<br>_99 KEINE ANTWORT

63.	S067	Q52. Hat Ihr Vater jemals Ekzeme, Hautallergien oder allergischen Schnupfen wie z.B. Heuschnupfen gehabt?<br><br>_1 nein<br>_2 ja<br>_98 WEISS NICHT<br>_99 KEINE ANTWORT
64.	S070	Q53. Haben Sie, bevor Sie 5-jährig waren, eine ernsthafte Entzündung der Atemwege gehabt? (gilt nicht: Schnupfen und Erkältung)<br><br>_1 nein<br>_2 ja (z.B. Lungenentzündung oder Bronchitis)<br>_98 WEISS NICHT<br>_99 KEINE ANTWORT
65.	T_H02040	Q54. Haben Sie schon einmal mindestens ein Jahr lang geraucht?<br><br>(„Ja“ heisst mindestens 20 Zigarettenpackungen oder 360g Tabak im ganzen Leben ODER: mindestens 1 Zigarette pro Tag, oder eine Zigarre pro Woche für ein Jahr).<br><br>_1 nein → *gehen Sie bitte zu Frage T_H02150*<br>_2 ja<br>_98 WEISS NICHT<br>_99 KEINE ANTWORT
66.	T_H02050	Q55. In welchem Alter haben Sie angefangen, regelmässig zu rauchen?<br><br>____________ (Zahl einfüllen)<br>_98 WEISS NICHT<br>_99 KEINE ANTWORT

67.	T_H02060	Q56. Rauchen Sie zur Zeit (im letzten Monat)?<br><br>_1 nein → *gehen Sie bitte zu Frage T_H02105*<br>_2 ja<br>_98 WEISS NICHT<br>_99 KEINE ANTWORT
68.	T_H02070	Q57. Wie viel rauchen Sie jetzt im Durchschnitt?<br><br>Anzahl Zigaretten pro Tag<br><br>________ (Zahl einfüllen)<br>_98 WEISS NICHT<br>_99 KEINE ANTWORT
69.	T_H02105	Q58. In welchem Alter haben Sie aufgehört zu rauchen?<br><br>____________ (Zahl einfüllen)<br>_98 WEISS NICHT<br>_99 KEINE ANTWORT

70	T_H02110	Q59. In der gesamten Zeit, in der Sie rauchten, haben Sie durchschnittlich wie viel geraucht?<br><br>Anzahl Zigaretten pro Tag<br><br>________ (Zahl einfüllen)<br>_98 WEISS NICHT<br>_99 KEINE ANTWORT
71.	T_H02150	Q60. Sind Sie in den letzten 12 Monaten regelmässig Tabakrauch ausgesetzt gewesen?<br><br>(regelmässig heisst, an den meisten Tagen oder Nächten)<br><br>_1 nein → *gehen Sie bitte zu Frage S051*<br>_2 ja<br>_98 WEISS NICHT<br>_99 KEINE ANTWORT
72.	T_H02170	Q61. Rauchen an Ihrem Arbeitsplatz andere Personen regelmässig?<br><br>_1 nein<br>_2 ja<br>_98 WEISS NICHT<br>_99 KEINE ANTWORT
73.	S051	Q62. Als Sie ein Kind waren, hat Ihre Mutter jemals regelmässig geraucht? *(bis 14 jährig)*<br>_1 nein<br>_2 ja<br>_98 WEISS NICHT<br>_99 KEINE ANTWORT

74	T_H01720	Q63. Welche Aussage beschreibt Ihre Wohnsituation am besten? Ich wohne ...<br><br>_1 im Stadt/Dorfzentrum an stark befahrener Strasse<br>_2 im Stadt/Dorfzentrum an wenig bis mässig befahrener Strasse<br>_3 im Aussenquartier/am Dorfrand an mässig bis stark befahrener Strasse<br>_4 im Aussenquartier/am Dorfrand an wenig befahrener Strasse<br>_5 im alleinstehenden Haus auf dem Land<br>_98 WEISS NICHT<br>_99 KEINE ANTWORT
75.	T_H01730	Q64. Wie gross ist werktags das Verkehrsaufkommen auf der Strasse, an welcher Sie wohnen?<br><br>_1 Stark befahrene Strasse/ununterbrochener Verkehrsfluss<br>_2 Mässig befahrene Strasse/viele Autos fahren vorbei<br>_3 Wenig befahrene Strasse/nur ab und zu ein paar Autos<br>_98 WEISS NICHT<br>_99 KEINE ANTWORT

76.	T_H01740	Q65. Wie oft fahren an Wochentagen Lastwagen durch die Strasse, an welcher Sie wohnen?<br><br>_1 nie<br>_2 selten<br>_3 öfter am Tag<br>_4 fast den ganzen Tag<br>_98 WEISS NICHT<br>_99 KEINE ANTWORT
