# Supplementary material for: Impacts of highway traffic exhaust in alpine valleys on the respiratory health in adults: a cross-sectional study
Source: Environ Health. 2011 Mar 4;10:13. doi: 10.1186/1476-069X-10-13 (PMC3059289; doi:10.1186/1476-069X-10-13)
Supplement: Additional file 2 — Definitions of reported respiratory symptoms. Text document that provides precise definitions of respiratory health outcomes. [file 1476-069X-10-13-S2.DOC]

**Additional File 2 – Definitions of reported respiratory symptoms**

Respiratory symptoms were defined based on the following specific groups of question(s) each of which had to be answered affirmatively: *Wheezing*: "Have you had wheezing or whistling in your chest at any time in the last 12 months?" *Wheezing with breathing problems*: Wheezing and an affirmative answer to “Have you had breathing problems when you had this wheezing or whistling?” *Wheezing apart from colds*: Wheezing and an affirmative answer to “Have you had this wheezing or whistling when you did not have a cold?” *Regular cough:* Affirmative answer to “Do you usually cough first thing in the morning?” and/or “Do you usually cough during the day, or at night?”

*Regular phlegm*: Affirmative answer to “Do you usually bring up any phlegm from your chest first thing in the morning?” and/or “Do you usually bring up any phlegm from your chest during the day, or at night?” *Chronic cough:* Regular cough and an affirmative answer to "Do you cough like this on most days for as much as three months each year?” and an answer of "≥ 2" to the question "For how many years?". *Chronic phlegm:* Regular phlegm and an affirmative answer to the question "Do you bring up phlegm like this on most days for as much as 3 months each year?” and an answer of "≥ 2" to the question "For how many years?".
