# Supplementary material for: Impacts of highway traffic exhaust in alpine valleys on the respiratory health in adults: a cross-sectional study
Source: Environ Health. 2011 Mar 4;10:13. doi: 10.1186/1476-069X-10-13 (PMC3059289; doi:10.1186/1476-069X-10-13)
Supplement: Additional file 3 — Sensitivity and supplementary analyses. This document provides a more complete version of Table 1, additionally containing the n's of the different categories (Table S1). In addition, it contains six tables with results from supplementary analyses on the association between reported respiratory symptoms and "living within 200 m of a highway": Table S2 reports adjusted odds ratios for reported respiratory symptoms in participants with and without asthma. Table S3 reports odds ratios adjusted only for community but not for individual factors. Table S4 shows odds ratios unadjusted for doctor's diagnosed asthma. Table S5 shows the results obtained after exclusion of non-Swiss citizens. Table S6 reports odds ratios for participants living more than 50 m from a major road. Finally, Table S7 reports odds ratios adjusted for satisfaction with air quality. [file 1476-069X-10-13-S3.DOC]

**Additional File 3 – Sensitivity and supplementary analyses**

**Additional File 3, Table S1.** Characteristics of the study population and a sub-group with allergy by residential distance to highway. The number of actual persons are displayed in brackets.

|  | **All participants**  **(total group)** | | **Low exposure** | | **High exposure** | | **Low exposure** | | **High exposure** | |
| --- | --- | --- | --- | --- | --- | --- | --- | --- | --- | --- |
|  |  |  | **(>200 m of a highway)** | | **(≤200 m of a highway)** | | **Participants with allergic  rhinitis or hay fever** | | | |
| n | 1581 | | 1384 | | 197 | | 288 | | 42 | |
| Men (%) | 46.5 | *(735)* | 45.6 | *(631)* | 52.8 | *(104)* | 46.9 | *(135)* | 57.1 | *(24)* |
| Age, mean | 41.7 |  | 41.9 |  | 40.2 |  | 37.7 |  | 36.5 |  |
| Primary school education only (%) | 5.7 | *(90)* | 5.7 | *(79)* | 5.6 | *(11)* | 3.8 | *(11)* | 2.4 | *(1)* |
| Swiss nationality (%) | 88.2 | *(1394)* | 87.6 | *(1212)* | 92.4 | *(182)* | 88.2 | *(254)* | 90.5 | *(38)* |
| Body Mass Index, mean | 24.4 |  | 24.4 |  | 24.5 |  | 23.7 |  | 24.2 |  |
| Maternal atopy (%) | 12.4 | *(196)* | 12.9 | *(179)* | 8.6 | *(17)* | 25.7 | *(74)* | 9.5 | *(4)* |
| Early childhood respiratory infection (%) | 7.7 | *(121)* | 7.4 | *(102)* | 9.6 | *(19)* | 11.5 | *(33)* | 11.9 | *(5)* |
| ETS exposure (%) | 27.3 | *(431)* | 27.0 | *(374)* | 28.9 | *(57)* | 30. | *(87)* | 31.0 | *(11)* |
| ETS exposure at workplace (%) | 12.5 | *(197)* | 12.6 | *(174)* | 11.7 | *(23)* | 13.5 | *(39)* | 11.9 | *(5)* |
| Occupational exposure, current (%) | 12.7 | *(200)* | 12.5 | *(173)* | 13.7 | *(27)* | 11.1 | *(32)* | 16.7 | *(7)* |
| Never smoker (%) | 57.2 | *(905)* | 57.6 | *(797)* | 54.8 | *(108)* | 63.9 | *(184)* | 71.4 | *(30)* |
| Current smoker (%) | 22.6 | *(357)* | 22.3 | *(308)* | 24.9 | *(49)* | 17.7 | *(51)* | 11.9 | *(5)* |
| Former smoker (%) | 20.2 | *(319)* | 20.2 | *(279)* | 20.3 | *(40)* | 18.4 | *(53)* | 16.7 | *(7)* |
| Smoked cigarettes (packyears), mean | 6.6 |  | 6.6 |  | 6.7 |  | 4.3 |  | 6.0 |  |
| Wheezing with breathing problems (%) | 4.7 | *(74)* | 4.5 | *(62)* | 6.1 | *(12)* | 9.7 | *(28)* | 11.9 | *(5)* |
| Wheezing without colds (%) | 4.9 | *(77)* | 4.6 | *(63)* | 7.1 | *(14)* | 7.3 | *(21)* | 11.9 | *(5)* |
| Regular phlegm (%) | 10.5 | *(166)* | 10.3 | *(143)* | 11.7 | *(23)* | 13.2 | *(38)* | 16.7 | *(7)* |
| Regular cough (%) | 15.0 | *(236)* | 14.9 | *(206)* | 15.2 | *(30)* | 18.9 | *(54)* | 21.4 | *(9)* |
| Chronic cough (%) | 5.8 | *(91)* | 5.4 | *(75)* | 8.1 | (16) | 6.6 | *(19)* | 16.7 | *(7)* |
| Chronic cough or chronic phlegm (%) | 6.7 | *(106)* | 6.5 | *(90)* | 8.1 | (16) | 9.4 | *(27)* | 16.7 | *(7)* |
| Doctor diagnosed asthma (%) | 8.9 | *(140)* | 9.1 | *(126)* | 7.1 | (14) | 20.8 | *(60)* | 14.3 | *(6)* |
| Allergic rhinitis or hay fever (%) | 20.9 | *(330)* | 20.8 | *(288)* | 21.3 | (42) |  |  |  |  |

**Additional File 3, Table S2. Adjusted odds ratios and 95% confidence intervals for reported respiratory symptoms associated with "living within 200 m of a highway" as traffic exposure proxy. Participants with and without doctor diagnosed asthma.**

| **Symptoms** | **Participants with or without doctor diagnosed asthma**  **n = 1566-1568** | | |
| --- | --- | --- | --- |
|  | with doctor diagnosed asthma | without doctor diagnosed asthma | p-value for interaction |
| Wheezing with breathing problems | 5.50 (0.95-31.7) | 2.24 (0.85-5.95) | 0.34 |
| Wheezing without colds | 4.80 (0.92-25.2) | 2.84 (1.11-7.27) | 0.54 |
| Regular cough | 2.96 (0.78-11.2) | 1.25 (0.65-2.40) | 0.20 |
| Regular phlegm | 1.25 (0.27-5.80) | 1.19 (0.59-2.40) | 0.94 |
| Chronic cough | 7.79 (1.03-22.2) | 2.62 (1.03 -6.67) | 0.43 |
| Chronic cough or phlegm | 3.37 (0.75-15.3) | 2.26 (0.92 -5.54) | 0.59 |

**Additional File 3, Table S3. Adjusted odds ratios and 95% confidence intervals for reported respiratory symptoms associated with "living within 200 m of a highway" as traffic exposure proxy, from a model including only community but no individual factors.**

| **Symptoms** | **Adjusted only for community**  n = 1566-1568 | **Adjusted for individual factors and community**  n = 1566-1568 |
| --- | --- | --- |
| Wheezing with breathing problems | 1.54 (0.69-3.43) | 2.64 (1.07-6.48)* |
| Wheezing without cold | 2.07 (0.93-4.61) | 3.10 (1.27-7.55)* |
| Regular cough | 1.30 (0.72-2.35) | 1.36 (0.72-2.56) |
| Regular phlegm | 1.14 (0.60-2.19) | 1.19 (0.60-2.38) |
| Chronic cough | 2.29 (1.00-5.23)* | 2.88 (1.17-7.05)* |
| Chronic cough or phlegm | 1.85 (0.84-4.07) | 2.40 (1.01-5.70)* |

*p-value <0.05

**Additional File 3, Table S4. Adjusted odds ratios and 95% confidence intervals for reported respiratory symptoms associated with "living within 200 m of a highway" as traffic exposure proxy. Adjusted for all covariates other than doctor’s diagnosed asthma.**

| **Symptoms** | **Total group**  n = 1566-1568 | **Participants with or without hay fever or allergic rhinitis**  n = 1561-1563 | |
| --- | --- | --- | --- |
|  |  | **with hay fever or allergic rhinitis** | **without hay fever or allergic rhinitis** |
| Wheezing with breathing problems | 1.82 (0.78-4.21) | 1.76 (0.50-6.14) | 1.75 (0.65-4.72) |
| Wheezing without colds | 2.13 (0.92-4.92) | 2.74 (0.79-9.51) | 1.88 (0.73-4.83) |
| Regular cough | 1.24 (0.67-2.32) | 1.65 (0.63-4.30) | 1.18 (0.60-2.33) |
| Regular phlegm | 1.11 (0.56-2.20) | 1.26 (0.44-3.60) | 1.03 (0.49-2.18) |
| Chronic cough | 2.34 (0.98-5.59) | 5.18 (1.57-17.10) | 1.64 (0.61-4.39) |
| Chronic cough or phlegm | 1.90 (0.82-4.37) | 3.32 (1.06-10.40) | 1.45 (0.56-3.74) |

*p-value <0.05

**Additional File 3, Table S5. Adjusted odds ratios and 95% confidence intervals for reported respiratory symptoms associated with "living within 200 m of a highway" as traffic exposure proxy, among Swiss citizens only**.

| **Symptoms** | **Subsample of Swiss citizens** | **n** |
| --- | --- | --- |
| Wheezing with breathing problems | 2.44 (0.94-6.34) | 1381 |
| Wheezing without colds | 2.61 (1.01-6.72)* | 1381 |
| Regular cough | 1.60 (0.83-3.07) | 1379 |
| Regular phlegm | 1.36 (0.66-2.77) | 1381 |
| Chronic cough | 3.34 (1.31-8.51)* | 1380 |
| Chronic cough or phlegm | 2.73 (1.12-6.66)* | 1380 |

*p-value <0.05

**Additional File 3, Table S6. Adjusted odds ratios and 95% confidence intervals for reported respiratory symptoms associated with "living within 200 m of a highway" as traffic exposure proxy, after exclusion of persons living within 50m of a major road**.

| **Symptoms** | **Participants living more than 50m of a main road** | **n** |
| --- | --- | --- |
| Wheezing with breathing problems | 1.88 (0.73-4.89) | 1127 |
| Wheezing without colds | 2.39 (0.92-6.24) | 1127 |
| Regular cough | 1.58 (0.80-3.12) | 1125 |
| Regular phlegm | 1.41 (0.67-2.98) | 1127 |
| Chronic cough | 2.99 (1.14-7.88)* | 1126 |
| Chronic cough or phlegm | 2.41 (0.95-6.11) | 1126 |

*p-value <0.05

**Additional File 3, Table S7. Adjusted odds ratios and 95% confidence intervals for reported respiratory symptoms associated with "living within 200 m of a highway" as traffic exposure proxy, from a model taking into account satisfaction with air quality**.

| **Symptoms** | **Core model  +  satisfaction with air quality ^1^**  n=797^2^ | **Core model**  n = 797^2^ |
| --- | --- | --- |
| Wheezing with breathing problems | 2.28 (0.54-9.66) | 2.11 (0.51-8.73) |
| Wheezing without cold | 2.32 (0.51-10.5) | 2.51 (0.61-10.4) |
| Regular cough | 0.99 (0.41-2.41) | 1.07 (0.45-2.53) |
| Regular phlegm | 1.05 (0.37-2.95) | 1.17 (0.43-3.22) |
| Chronic cough | 2.22 (0.56-8.78) | 2.43 (0.66-8.98) |
| Chronic cough or phlegm | 2.16 (0.58-7.98) | 2.34 (0.66-8.28) |

^1^Satisfaction with air quality was assessed on an integer scale from -5 to 5. To account for non-linearities in the relationship, a third order polynomial of this variable was used in the model.
^2^Satisfaction with air quality was provided by about 50% of our study participants in a previous survey.
